# Unprocessed snRNAs Are a Prognostic Biomarker and Correlate with a Poorer Prognosis in Colorectal Cancer

**DOI:** 10.3390/cancers16132340

**Published:** 2024-06-26

**Authors:** Víctor Escrich, Cristina Romero-Aranda, Rosario López, María de Toro, Ángela Metola, Begoña Ezcurra, Eva Gómez-Orte, Juan Cabello

**Affiliations:** 1 Oncology Area, Center for Biomedical Research of La Rioja—CIBIR, 26006 Logrono, Spain; vescrich@riojasalud.es (V.E.); craranda@riojasalud.es (C.R.-A.); mthernando@riojasalud.es (M.d.T.); ametola@riojasalud.es (Á.M.); bezcurra@riojasalud.es (B.E.); 2 Scientific Computing Group (GRUCACI), University of La Rioja, 26006 Logroño, Spain; rosario.lopez@unirioja.es

**Keywords:** *INTS6*, snRNA processing, colorectal cancer, prognosis

## Abstract

**Simple Summary:**

Integrator-complex deregulation is involved in 8.3% of colorectal cancer cases. Lack of Integrator-complex function, measured by an increased level of unprocessed snRNA, is a prognostic biomarker and correlates with a poorer prognosis in colorectal cancer. Our results show that lack of Integrator-complex function (measured as level of snRNA processing) and not necessarily its expression level correlates with a poorer prognosis in colorectal-cancer patients. Population screening, combined with early typing of tumors, appears to be the most efficient way to increase patient survival.

**Abstract:**

The human Integrator complex is a set of 15 subunits that mediates processing of small nuclear RNAs (snRNAs), and which later participates in splicing messenger RNAs (mRNAs). In addition, it controls the pause and release of RNA polymerase II (RNA pol II) at specific gene promoters in response to growth factors. Mutations in Integrator-complex subunit 6 (*INTS6*) are associated with different types of tumors. However, the *INTS6* gene product does not have a significant prognostic value as a biomarker for tumor progression. Here we show that Integrator-complex deregulation is involved in 8.3% of the colorectal cancer cases diagnosed from the population screen carried out in La Rioja (Spain) from the years 2017 to 2019. Lack of Integrator-complex function, measured by an increased level of unprocessed snRNA, is a prognostic biomarker and correlates with a poorer prognosis in colorectal-cancer patients. The transcriptomic profile of all analyzed colorectal tumors shows a strong alteration of the metabolic state of tumor cells, which compromises standard energy production through mitochondrial respiration, known as the Warburg effect. Furthermore, there is a significant upregulation of genes involved in extracellular matrix organization and collagen rearrangement. This is consistent with tissue reorganization in a growing tumor forming a polyp. Crossing the molecular data generated in this study with the follow-up of patients from population screening indicates that population screening combined with early typing of tumors appears to be the most efficient way to increase patient survival.

## 1. Introduction

Colon cancer is the second leading cause of cancer-related deaths worldwide. In 2020, more than 1.9 million new cases of colorectal cancer and more than 930,000 deaths due to colorectal cancer were estimated to have occurred worldwide [1].

Colorectal cancer is a type of cancer that affects the colon (large intestine) or rectum. The risk of colorectal cancer increases with age. Most cases affect people over 50 years old. Common symptoms include diarrhea, constipation, blood in the stool, abdominal pain, unexplained weight loss, fatigue, and low iron levels. Many people will not have symptoms in the early stages of the disease. The risk of colorectal cancer can be reduced by eating a healthy diet, staying physically active, not smoking tobacco and limiting alcohol consumption. Regular screenings are crucial for early detection [2]. In fact, incidence rates of colorectal cancer have been decreasing in high-income countries, largely as a result of effective screening programs [3]. The most common screening method is the fecal occult blood test (FOBT). The FOBT detects hidden blood in the stool, which can be an indicator of colorectal cancer or polyps. If blood or abnormal findings are detected in the stool, further diagnostic procedures, such as colonoscopy, are usually recommended to confirm the presence of colorectal cancer or polyps [4].

Diagnostic methods for colorectal cancer include physical examination, imaging (such as abdominal ultrasound, computed tomography scans, and magnetic resonance imaging), examination of the inside of the colon using colonoscopy or sigmoidoscopy, taking a sample of tissue (biopsy) for histopathological examination, and molecular testing to identify specific gene mutations or biomarkers to guide the best treatment option [2].

Treatments for colorectal cancer are based on type and progression of the tumor and the person’s medical history. The primary treatment for early-stage disease (tumor limited to the bowel or local lymph nodes, with no metastatic dissemination to distant organs) is surgical removal of the tumor and nearby lymph nodes. In advanced disease, the primary treatment approach involves chemotherapy and immunotherapy. In some cases, surgery may be recommended for metastatic colorectal cancer to remove tumors that are causing symptoms or blocking the intestine. After treatment, regular follow-up visits and monitoring are essential to detect for any signs of recurrence or new cancer [5,6].

The prognosis for colorectal cancer varies depending on the stage at diagnosis. Early-stage cancers have higher overall survival rates (defined as the percentage of patients who survive the cancer during a specific period of time) than advanced-stage cancers. Timely diagnosis, appropriate treatment, and regular follow-up care are important for improving survival rates and quality of life [5,6].

Over the last two decades, Integrator complex and especially Integrator subunit 6 (*INTS6*/OMIM 604331) dysregulation have been established to be involved in several types of tumors, such as prostate cancer [7,8,9], esophageal squamous cell carcinoma [10], nasopharyngeal carcinoma [11], hepatocellular carcinoma [12,13] or lung carcinoma [14] among others. This suggests that all these disparately located cancers share a common mechanism of tumor transformation as a consequence of Integrator-complex dysfunction.

The Integrator complex is evolutionary well-conserved from nematodes to humans [15,16], indicating that it plays a central role in the cell biology. It has been described as a set of 15 proteins in humans [15,17,18,19] that binds to RNA pol II and is responsible for snRNA processing during their transcription. Mature snRNAs then form part of the spliceosome for mRNA processing [20]. In addition to this function, Integrator complex also plays a role in pause and release of the RNA pol II at the promoter of specific genes [21,22] and DNA repair [23,24], and in some organisms, *INTS6* controls mitochondrial structure and function [25,26]. Given the multiple functions in which the Integrator complex participates, tumors that present mutations in Integrator subunits show global effects on the cell’s biology, altering key functions such as genomic stability and DNA repair, mitochondrial function, general gene expression and snRNA processing. A major consequence of Integrator mutation is the readthrough of the RNA pol II downstream of the snRNA loci [15,18] Consequently, long RNAs derived from unprocessed snRNAs are generated in the cells. In order to delve deeper into the functional characterization of tumors caused by defective Integrator, it is first necessary to establish a rapid and sensitive system for identifying their defects.

In this work, we analyze the impact that Integrator dysfunction has on colorectal tumors. To this end, we measure the level of Integrator subunit 6 mRNA and unprocessed snRNAs in a cohort of colorectal tumor samples and asses its value as a diagnostic and prognostic marker for those tumors. We conclude that snRNA processing may be a good biomarker for patient survival.

## 2. Materials and Methods

### 2.1. Patient Selection and Tissue Samples

Biopsy samples were obtained from a cohort of 90 patients showing different degrees of illness. Ten healthy biopsies were selected as control. All samples came from the San Pedro Hospital’s Digestive System Department (La Rioja, Spain). Pathological samples were obtained from lesions with suspected tumor origin observed during diagnostic colonoscopies performed between 2017 and 2019. In all cases, these samples were collected and processed with the informed consent of the patients. The samples from these biopsies were divided for routine histological analysis in the Anatomic Pathology Service and RNA extraction. Control samples came from biopsies of healthy mucosal tissue taken from a region close to the lesion in patients diagnosed with colorectal cancer of this study. This prevents biases related to the patient’s age and sex. Control samples were collected during the same exploration. The indication for having a colonoscopy is the same in all cases (preventive program for colorectal cancer, symptoms associated with the colon or radiological findings suggestive of colorectal pathology). The Mann–Whitney U test yielded a z-score of 2.49326 and a corresponding *p* value of 0.013. At a significance level of 0.01, the obtained *p* indicates that the difference in ages between the control group and the patients is not statistically significant. In other words, at a significance level of 0.01, the age distribution in control and patient samples are similar.

### 2.2. RNA Extraction

RNA extraction was performed with QIAGEN RNeasy^®^ Kit (QIAGEN GmbH, Hilden, Germany) following the instructions for purification of total RNA from animal cells from the manufacturer. Briefly, samples were collected in 350 µL RLT buffer containing β-mercaptoethanol and quickly frozen. Homogenization of the tissue was performed using a conventional Polytron^®^ Rotor-Stator homogenizer (Kinematica AG, Malters, Switzerland) pre-chilled with liquid nitrogen.

### 2.3. cDNA Synthesis

RNA was treated with DNase to eliminate any DNA contamination. In each sample, a total reaction of 10 µL contained 400 µg RNA, 1 µL RQ1 RNase-Free DNase (Promega Corp., Madison, WI, USA), 1× RQ1 DNase 10X Reaction Buffer and DEPC water. The reaction was incubated for 30 min at 37 °C, stopped by adding 1 µL Stop solution (Promega Corp., Madison, WI, USA) and incubated for 10 min at 65 °C. cDNA synthesis was performed using SuperScript™ III First-Strand Synthesis System for RT-PCR (Invitrogen-Life Technologies Corp., Paisley, UK) with random hexamer primers in a GeneAmp PCR System 9700, Applied Biosystems machine (Thermo Fisher Scientific, Waltham, MA, USA).

### 2.4. Semiquantitative PCR

*INTS6* (Gene ID: 26512. Chromosome 13, NC_000013.11) RNA expression was analyzed using the primers *INTS6*Fw: 5′-ACAAGGAGACTGGAATAAAAGTCC-3′ and *INTS6*Rv 5′-TAAACTGCCTTGCACATGCT-3′. A total of 1 µL of cDNA was used for PCR amplification (GeneAmp PCR System 9700, Applied, Foster City, CA, USA) and reactions were optimized to 94 °C for 2 min, 38 amplification cycles at 94 °C for 30 s, 59 °C annealing temperature for 1 min, 72 °C for 1 min, and a final extension of 10 min at 72 °C. *U2* processing was analyzed using the following primers in the *U2* snRNA pseudogene RN*U2*-69P. (Gene ID: 106481656. Chromosome 18, NC_000018.10): *U2*69Fw: 5′-CAAGGAGCTGAAAGGCACTGA-3′ and *U2*69Rv: 5′-GACCTGTGCTTTCTGGGGTAG-3′. Glyceraldehyde 3-phosphate dehydrogenase (*GAPDH*. Gene ID: 2597. Chromosome 12, NC_000012.12) was used as a housekeeping reference gene. *GAPDH* expression was analyzed using the primers *GAPDH*Fw 5′-AAATCCCATCACCATCTTCC-3′ and *GAPDH*Rv: 5′-GACTCCACGACGTACTCAGC-3′. PCR amplification for *U2* and *GAPDH* were optimized to 94 °C for 2 min, 38 amplification cycles for *U2* and 30 for *GAPDH* at 94 °C for 30 s, 59 °C annealing temperature for 1 min, 72 °C for 15 s, and a final extension of 10 min at 72 °C. A total of 7 µL of the amplified products was resolved on 1.5% agarose gels, visualized by SYBR™-safe staining (Invitrogen-Life Technologies, Carlsbad, CA, USA) and documented using the Bio-Rad Universal Hood II-GelDoc System, 1D Analysis Software: Quantity One 4.6.3, as TIFF digital images.

PCR products were quantified by measuring gel band intensity using ImageJ software (v1.52a) [27]. GelDoc images were stored as TIFF files. On the gel image, a region of a fixed area covering the size of the band on the gel was drawn using the “rectangular selection tool”. This region was moved over the different bands to measure the optical density of each. The optical density of the gel background was measured and subtracted from the value obtained for each band. In addition, we applied a correction factor based on the *GAPDH* loading control corresponding to each of the samples. The *GAPDH* band whose intensity was the highest was taken as a reference. Its value was divided by the intensity of each of the other samples to determine the correction factor that had to be applied to each one. Mean and standard deviations of the optical density values of the control samples were obtained to define whether the *U2* snRNA and *INTS6* mRNA levels in the tumor samples were high (greater than the mean + SD), medium (between the mean ± SD) or low (less than mean − SD).

### 2.5. Transcriptomic Analysis

Global gene expression profiles from healthy individuals and patients were analyzed at the Genomics and Bioinformatics Platform of the CIBIR, as previously described [16]. Briefly, sequencing libraries were prepared by following the Illumina Stranded Total RNA Prep with Ribo-Zero Plus (Illumina Inc., San Diego, CA, USA) protocol from 100 ng of the total extracted RNA. All libraries were run in a HiSeq1500 PE100 lane. After read quality analysis, trimming, and read mapping against the reference genome, the expression analysis was performed using DESeq2 (v1.34.0) [28] and EdgeR (v3.36.0) [29] algorithms through the SARTools (v1.7.4) [30] pipeline. Genes were scored as differentially expressed genes (DEGs) if their expression, compared to the control, met the following parameters: Padj < 0.05, Up: log2FC > 0, Down: log2FC < 0. The reference genome GRCh38 (patch 105) was obtained from the ENSEMBL database (https://www.ensembl.org/info/data/ftp/index.html, accessed on 3 April 2024). The up- and downregulated genes were analyzed in order to find enriched GO-terms, KEGG and Reactome pathways by using the clusterProfiler R-package (v4.10.1) [31,32]. GO-term enrichment was ordered by their adjusted *p* value, listing first those which were statistically more significant.

### 2.6. Patient Monitoring 

Patients were monitored with different follow-up guidelines depending on the disease stage. For patients in stage I, surveillance consisted of an annual colonoscopy. If advanced adenoma was detected, the colonoscopy was repeated after one year and if not, it was repeated after three years and subsequently after five years. For patients in stages II and III, surveillance consisted of anamnesis, physical examination and CEA (carcinoembryonic antigen) testing every 3 months for 2 years, and then, every 6 months for 5 years. In addition, these patients underwent annual thoracic–abdominal–pelvic CT (Computed Axial Tomography) for 5 years and an annual colonoscopy. For patients in stage IV, surveillance consisted of anamnesis, physical examination and CEA testing every 3 months for 2 years, and then every 6 months for 5 years. These patients also underwent thoracic–abdominal–pelvic CT every 3–6 months for 5 years and an annual colonoscopy.

### 2.7. Statistical Analysis

Statistical computations were performed using the R programming language (version 4.0.5) within the RStudio integrated development environment (version 1.4.1106) [33], and specific libraries such as plotly (version 4.9.3) [34], heatmaply (version 1.2.1) [35], ggcorrplot (version 0.1.3) [36] were employed. Correlation of the variables was determined using the Spearman correlation analysis. The statistical significance of the correlation was determined using Student’s *t* distribution. This analysis allowed us to identify variables that had a strong correlation and quantify their influence on the target variable. All statistical tests were two-tailed and *p* < 0.05 was considered statistically significant. A Kaplan–Meier risk and survival analysis was then performed to investigate the occurrence of events of interest (in this case, survival) over time. Survival (version 3.2.11) [37], survminer (version 0.4.9) [38], ggplot2 (version 3.3.3) [39] and ggpubr (version 0.4.0) [40] were used as libraries for computing survival analyses and for summarizing and visualizing the results. Finally, univariate and multivariate nonparametric Cox analyses were performed to generate a predictive model for time-to-event wait data. While univariate Cox regression assessed the relationship between any clinicopathological predictor and survival outcome, multivariate Cox regression allowed us to extend this analysis by considering multiple predictors simultaneously, thereby capturing the combined effects of various factors on survival.

## 3. Results

### 3.1. Selection of a Set of Samples from Patients with Different Types of Colorectal Tumors

To study the effect that deregulation of the Integrator complex and snRNA processing have on colorectal cancer, we analyzed a cohort of 90 patients showing different degrees of illness, and 10 healthy individuals who served as controls. Patients were selected from the San Pedro Hospital’s Digestive System Department (La Rioja, Spain) based on the appearance of a lesion suspected to be of tumor origin during a diagnostic colonoscopy. The study was run from 2017 to 2019.

To evaluate the degree to which different types of tumors were represented in the selected cohort, we studied several clinicopathological characteristics of the analyzed tumors (Table 1). The group of patients included 54 males (60%) and 36 females (40%); 43 patients (47.8%) were less than 75 years old and 47 (52.2%) were more than 75 years old. This age of 75 years is important, since it is the age from which individuals are no longer included in population screening analyses in Spain. Regarding the reason why a colonoscopy was requested for the patient, 26 patients (28.9%) came from population screening, 46 (51.1%) presented a clinical picture suggestive of neoplasm in the consultation and 18 (20%) presented a previous radiological image in computed axial tomography (CT), nuclear magnetic resonance (MRI) or positron emission tomography (PET/CT) compatible with colon thickening/neoplasm. Further analysis by anatomical location showed that 29 tumors (32.2%) were located in the proximal colon, 35 (38.9%) in the distal colon and 26 (28.9%) in the rectum. Regarding the tumor stage, 17 patients (18.9%) had a grade 1 tumor, 19 patients (21.1%) had a grade 2 tumor, 34 patients (37.8%) had a grade 3 tumor, and 20 patients (22.2%) had a grade 4 tumor. Finally, most of the diagnosed colorectal cancers presented a moderate degree of differentiation at diagnosis (81 of 90, 90%). Therefore, we concluded that the multiple tumors represented in the selected cohort allowed us to study the effect of snRNA processing and *INTS6* levels in different types of colorectal tumors.

### 3.2. Classification of Colorectal Tumors Based on INTS6 Levels and snRNA Processing

To ascertain the impact that Integrator-complex deregulation and snRNA processing have on colorectal cancer, we analyzed their levels in biopsies of colon tumor lesions suggestive of malignancy within the selected cohort of 90 colorectal-tumor patients and compared these to healthy controls.

**Figure 1:**
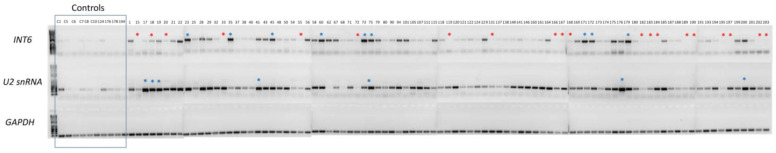
Analysis of *INTS6* mRNA levels and *U2*-69 snRNA processing in colorectal biopsy samples from healthy individuals who served as controls (blue box) vs. colorectal-tumor patients. Numbers correspond to biopsy sample identifiers. Semiquantitative RT-PCR of *INTS6* mRNA is shown in the top row. RT-PCR of the region immediately downstream of the *U2*-69 snRNA, as a measure of snRNA processing, is shown in the middle row. *GAPDH* mRNA levels (bottom row) are shown as normalization of the loading control. Red asterisks mark levels lower than the mean minus the standard deviation of the corresponding control samples and blue asterisks mark levels greater than the mean minus the standard deviation of the corresponding controls.

*INTS6* mRNA expression was measured by semiquantitative RT-PCR using internal primers from the gene sequence. snRNA processing was defined by measuring transcription in the region directly downstream of the *U2*-69 snRNA also using semi-quantitative PCR with a pair of primers in that region (as described in Material and Methods). *GAPDH* expression was used as an endogenous control for normalizing data (Figure 1). Mean ± standard deviation of expression in healthy control samples was used to define three categories of *INTS6* expression and snRNA processing: low, normal, and high expression. 

For *INTS6* expression, 16 samples (17.8%) from patients showed low levels of expression (lower than the mean minus the standard deviation in healthy individuals). A total of 30 samples (30.3%) showed normal levels (those included between the values of the mean ± the standard deviation in healthy individuals) and 44 samples (48.9%) showed high levels of *INTS6* expression (higher than the mean plus the standard deviation in healthy individuals). Similarly, 73 (81.1%) patient tumor samples showed normal snRNA processing (those included between the mean ± the standard deviation values of healthy individuals). A total of 17 patient tumors (18.9%) showed high levels of unprocessed snRNAs (higher than the mean plus the standard deviation of expression in healthy individuals). This group represents those samples which were defective on processing the snRNAs. Interestingly, we did not find any tumors with low levels of unprocessed snRNAs (lower than the mean plus the standard deviation of expression in healthy individuals), which would indicate more efficient snRNA processing than healthy cells. The absence of any tumors in this category indicates that snRNA processing is highly optimized in normal cells and neither a mutation nor deregulation of the Integrator complex can make it more efficient (Table 2).

### 3.3. Tumor Cluster in Different Transcriptional Categories Based on snRNA Processing and INTS6 mRNA Levels

To assess whether snRNA processing and *INTS6* mRNA levels affect the global gene expression profile of tumors, we analyzed the transcriptome of three biopsy samples selected from each of the six categories derived by combining snRNA processing (proper or defective) and *INTS6* expression (low, normal or high) data. In addition, we also included in the analysis samples from three biopsies from healthy individuals as controls. Raw sequence data generated in this study are available in the Gene Expression Omnibus (GEO) data repository (Accession number GSE261888).

**Figure 2:**
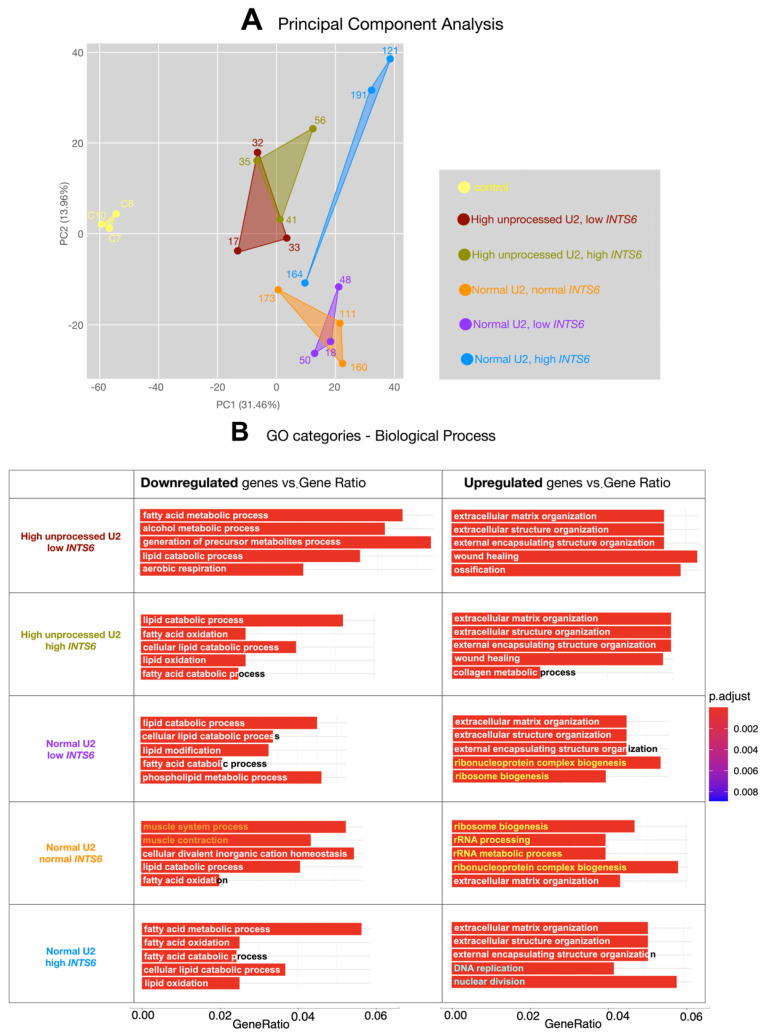
Transcriptomic analysis of the tumor categories based on *U2* snRNA processing and *INTS6* mRNA level. (**A**) Principal Component Analysis (PCA) plot based on DESeq2 regularized log2 transformation (rlog) data shows clusters according to the similarity of the transcriptional profile. Three samples from each tumor category are shown in the 2D plane, spanned by their first two principal components. Controls corresponding to biopsies from healthy individuals (yellow) grouped very closely, indicating very similar transcriptional profiles. Biopsy samples containing high levels of unprocessed *U2* snRNAs (brown and green) show a more similar transcriptional profile to those of controls than samples with normal levels of *U2* snRNA processing (orange, purple, and blue). Samples containing high levels of *INTS6* mRNA separate from those with low or normal levels of *INTS6*. Among all analyzed samples, those corresponding to normal processing of *U2* and high levels of *INTS6* (blue) show the most different transcriptional profile from those of healthy controls. (**B**) Gene ontology (GO) analysis of up- and downregulated gene biological processes (BP). GO of BP vs. number of genes within each category are shown as color bars, one bar per GO term. Bar length indicates the number of genes belonging to the different GO categories and color illustrates the statistical significance. Only the five most significant GO terms (adjusted *p* < 0.002, bars color-coded in red) are shown. All defined groups share common features such as downregulation of fatty acid metabolism or mitochondrial respiration and upregulation of genes involved in the organization of the extracellular matrix. However, those groups showing a more distant transcriptional profile from that of the control upregulate genes involved in ribosome biogenesis (highlighted with font color yellow), muscle contraction (highlighted with font color orange) or cell division (highlighted with font color cyan).

Gene expression data were normalized by a negative binomial distribution model using DESeq2 and EdgeR implementations. The global-expression profile similarity of all the samples was analyzed using a principal component analysis (PCA) plot (Figure 2A) generated with DESeq2 (v1.34.0) [28]. Very close clustering of the three controls reflects a highly uniform transcriptional signature on biopsy samples from healthy individuals and indicates that colon mucosa biopsy cells express a specific and well-defined set of genes with low variability among individuals. Thus, a colon mucosa biopsy appears to be a good system in which to study gene expression alterations. Differences in gene expression patterns in samples will likely be caused by tumor transformation rather than by individual variability. Strikingly, samples from categories that have high levels of unprocessed *U2* plot closer to healthy controls than samples with normal *U2* snRNA processing along the principal component 1 (PC1) axis in the PCA. In addition, samples containing high levels of *INTS6* mRNA separate from those with low or normal levels of *INTS6* along the PC2 axis. These data illustrate a trend in which tumors with defective *U2* snRNA processing (and which, therefore, accumulate unprocessed *U2*) show a more similar gene expression pattern to those of healthy controls than those tumors with normal processing of *U2*. Among all analyzed samples, those belonging to the category including normal processing of *U2* and high levels of *INTS6* show the most dissimilar transcriptional profile from those of healthy controls (Figure 2A). 

To gain insight into the transcriptional features of each category, we performed a gene ontology analysis (GO)-term enrichment of up- and downregulated gene biological processes (BPs) in each category. The three biopsy samples of each category were considered biological replicas for the statistical analysis of gene expression in that category. Quantitative analysis of differential expression was performed as described in materials and methods in [28,29,30]. The five most significant GO terms (adjusted *p* < 0.002) in each group are shown in Figure 2B (generated with the function “enrichGO” of the clusterProfiler R-package v4.10.1) [31,32].

No downregulated genes in any of the analyzed groups fall within multiple categories of GO biological processes or cellular compartments. Instead, in all cases, there is a remarkable downregulation of genes involved in processes such as lipid oxidation, aerobic respiration, or metabolism. These genes code for proteins whose location is enriched in mitochondria and cellular apical membrane compartments (Tables S1 and S2). These transcriptomic changes either cause or reflect a dramatic alteration in the metabolic state of tumor cells, compromising standard energy production by mitochondrial respiration, and suggest an alternative production of energy through cytosolic fermentation. This mechanism is known as the Warburg effect [41,42,43]. Interestingly, in addition to the mentioned metabolic changes, samples of tumors with normal *U2* snRNA processing and normal *INTS6* mRNA levels also show a remarkable downregulation of genes involved in muscle contraction (Figure 2B).

Parallel to this gene downregulation response, gene upregulation also shows a common set of biological processes enriched in all the analyzed groups. This upregulation mainly affects genes involved in extracellular matrix organization and collagen rearrangement. This is consistent with tissue reorganization in a growing tumor forming a polyp. In addition to this general feature, categories showing a more distant transcriptional profile from the control (such as those with normal processing of *U2* snRNAs and low or normal levels of *INTS6* mRNA) show upregulation of ribosome biogenesis. Finally, tumors with normal processing of *U2* snRNAs and a high level of *INTS6* mRNA (transcriptionally, the most different from those of the healthy control samples) show a high expression of genes involved in DNA replication and cell division. This could be indicative of a high proliferation ratio in those tumors (Figure 2B, Tables S1 and S2).

### 3.4. Unprocessed U2 snRNAs Level Correlates with Other Tumor Features

**Figure 3:**
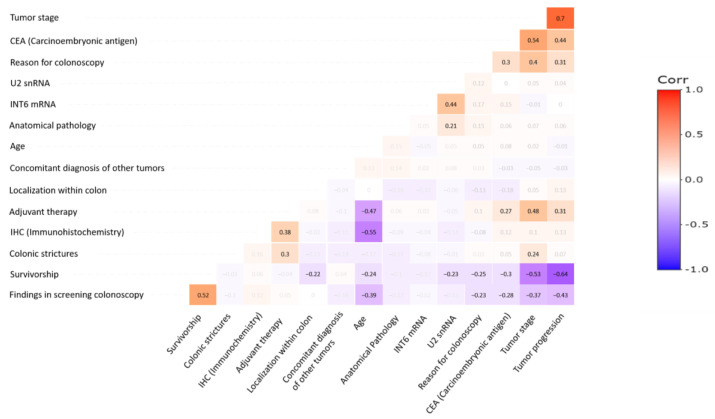
Spearman correlation matrix, highlighting rho significant coefficients. Coefficients with a *p* < 0.05 are shown sharply highlighted to indicate significance, while those with a *p* > 0.05 are blurred to indicate insignificance. The coefficients range from −1 to 1, indicating the strength and direction of the monotonic relationship between pairs of variables. Positive values signify a direct monotonic relationship, while negative values indicate an inverse monotonic relationship. Coefficients close to 1 or −1 denote a strong monotonic association, whereas coefficients near 0 suggest a weak or non-monotonic relationship. Each cell in the matrix represents the Spearman correlation coefficient between the corresponding pair of variables, providing insights into their mutual relationships. Thus, there is a negative correlation between levels of unprocessed *U2* and patient survival (*p* = 0.029, *rho* = −0.229). This indicates that lack of *U2* processing in the tumor correlates with lower patient survival. In addition, there is a positive correlation between levels of unprocessed *U2* and levels of *INTS6* mRNA (*p* = 0.0, *rho* = 0.436). This indicates that lack of *U2* processing in the tumor correlates with high levels of *INTS6* mRNA.

To evaluate the value of snRNA processing and *INTS6* mRNA levels as possible biomarkers for the diagnosis and prognosis of colorectal cancer, we performed a Spearman correlation analysis (Figure 3) with the rest of the clinicopathological variables of the tumors. Statistical computations were performed using the R programming language (version 4.0.5) within the RStudio integrated development environment (version 1.4.1106). The Spearman correlation coefficient is a statistical measure used to assess the relationship between two categorical or ordinal variables. To conduct the correlation analysis, the categorical variables were transformed into ordinal scales (Table S3). Specifically, each category within the variables was assigned to a numerical value representing its position in a meaningful order or ranking. This transformation ensured that the data maintained their inherent structure while enabling the use of Spearman’s rank correlation coefficient to effectively explore associations between variables (as described in [44,45]). The Spearman coefficient allows us to capture the direction and strength of the association. Figure 3 displays the Spearman correlation matrix, highlighting significant rho coefficients. The statistical significance of the correlation was determined using the Student’s t distribution, with degrees of freedom equal to the sample length minus two. Coefficients with a *p* value < 0.05 are shown sharply highlighted to indicate significance, while those with a *p* value > 0.05 are blurred to indicate insignificance. The complete tables containing all the coefficients and their corresponding *p* values are provided as Supplementary Material (Table S4). In addition, Table 1 encompasses all analyzed characteristics transformed into ordinal scales, and their association with *U2* expression levels stratified into medium and high categories. Each relationship includes the associated number of samples, the total sample size, the *p* value extracted from the correlation matrix, and the correlation coefficient.

Importantly, we observed a negative correlation between unprocessed *U2* snRNA and survival. Lack of snRNA processing and subsequent accumulation of unprocessed snRNAs in tumors significantly correlates with lower survival expectancy in patients (*p* = 0.029, *rho* = −0.229). In addition, there was a positive correlation between unprocessed *U2* snRNA and *INTS6* levels (*p* = 1 × 10^−9^, *rho* = 0.436). High levels of unprocessed *U2* snRNA are statistically more abundant in tumors with high levels of *INTS6* mRNA (12 of 44, 27.3%) than among those with low or medium levels of *INTS6* mRNA (5 of 46, 10.9%). This result suggests the existence of a feedback mechanism, in which *INTS6* expression could be induced upon lack of snRNA processing to revert the accumulation of unprocessed snRNAs. Moreover, we evaluated the relationship between tumor grade and levels of unprocessed *U2* snRNA. We observed that in the initial stages (I and II) there are fewer patients with high levels of unprocessed *U2* snRNA (5 out 36) than in more advanced stages (III and IV) (12 out 54), but this increase is not statistically significant (*p* = 0.594, *rho* = 0.050). Finally, we found no correlation between *U2* snRNA processing and other variables analyzed such as the reason for requesting the colonoscopy, evaluation of revision colonoscopies, endoscopic pattern presented by the neoplasm, presence of carcinoembryonic antigen (CEA) or age. With respect to the anatomical location of the tumors, high levels of unprocessed *U2* snRNA were uniformly distributed in all groups and appeared in five samples from tumors located in the proximal colon, seven in the distal colon, and five from rectal tumors.

Unlike *U2* snRNA processing, *INTS6* mRNA levels did not show significant correlation with any of the studied clinicopathological features of the tumors. This indicates that although the Int6 protein level has been described as a marker for certain tumors such as hepatocellular carcinomas or breast cancer, [13,46] *INTS6* mRNA level cannot be considered a biomarker. This suggests a mechanism in which *INTS6* might be post-transcriptionally deregulated in tumor cells, either translationally or at the level of protein stability. Alternatively, *INTS6* might be involved in tumors affecting other tissues, but not in colorectal cancer.

Regarding the other tumor features, we found that the presence of tumor markers (*p* = 0.005, *rho* = −0.302), high tumor stage (*p* = 0.0064 × 10^−4^, *rho* = −0.530), or advanced progression (*p* = 0.0087 × 10^−9^, *rho* = −0.641) have a very strong negative correlation with survival time, meaning that the more developed the tumor is, the lower the life expectancy of the patient. Along those lines, we found a strong positive correlation between tumor progression and tumor stage (*p* = 0.0031 × 10^−10^, *rho* = 0.7), and presence of tumor markers and tumor stage (*p* = 0.0068 × 10^−5^, *rho* = 0.54). This indicates that high-grade tumors have progressed more and express more tumor markers than tumors that were detected early. Other significant correlations are more related to medical practice than to tumor characteristics. Thus, age shows a negative correlation with the prescription of immunohistochemistry analysis or surgical procedures. The clinical goal is to prevent elderly patients from undergoing aggressive treatments wherein recovery is more harmful than the tumor itself. In these circumstances, palliative care is considered. Finally, performing revision colonoscopies shows a positive correlation with survival since they are only performed in surviving patients. These expected results serve as an internal control for the quality of the statistical analysis.

In conclusion, levels of unprocessed *U2*snRNAs (but not *INTS6* mRNA levels) may be a good biomarker for diagnosis/prognosis of colorectal tumors. 

### 3.5. Unprocessed U2 snRNA Level Impacts Survival

**Figure 4:**
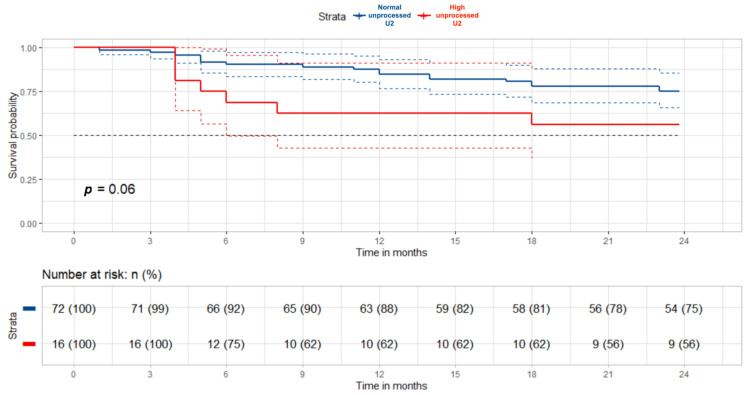
Kaplan–Meier survival analysis according to *U2* snRNA processing: Horizontal axis (*x*-axis) shows time in months and vertical axis (*y*-axis) shows the survival probability. Lines represent the survival curves of the two groups into which we have separated the samples: normal unprocessed *U2* snRNA levels (blue line) and high unprocessed *U2* snRNA levels (red line). At the start of the study (time zero), all the patients studied were alive and, therefore, the probability of survival was 1. After 6 months and onwards, the survival probability was approximately 20% lower for patients with high levels of unprocessed *U2* snRNAs compared to those with normal processing of *U2* snRNAs.

To assess the impact that high levels of unprocessed snRNAs in tumors have on patient survival, we performed a Kaplan–Meier analysis. We divided the patient cohort into two groups (those with normal processing of *U2* snRNAs and those with high levels of unprocessed *U2* snRNAs) and followed their survival throughout the duration of the study (24 months). We observed that six months after diagnosis, survival in the group that had high levels of unprocessed *U2* snRNA was consistently around 20% lower compared to the group with normal *U2* snRNA processing. A total of 47% of patients with tumors showing high levels of unprocessed *U2* snRNA died during the time the study lasted. In contrast, 28.7% of patients with tumors showing normal levels of unprocessed *U2* snRNA died during the same period (Figure 4).

To deconstruct the impact that unprocessed *U2* snRNA levels have on survival, not only at the global level, but under different patient clinicopathological conditions, we performed a multivariate Cox analysis. The univariate Cox analysis regarding survival did not indicate statistically significant association with *U2* snRNA processing (*p* = 0.16) (Table S5A). However, conducting a multivariate Cox model to simultaneously observe the influence of unprocessed *U2* levels, combined with multiple other clinicopathological conditions of patients revealed a significant impact of *U2* processing in combination with age, tumor progression and *INTS6* levels on patient survival (*p* of 0.047, log-rank test *p* = 7 × 10^−5^) (Table S5B). By considering the joint contribution of multiple factors, rather than assessing each variable in isolation, multivariate analysis produces more robust and clinically relevant prognostic models. We observed, for instance, that in all groups, high levels of unprocessed *U2* snRNA correlated with a worse prognosis (Figure S1). This indicates that snRNA processing per se constitutes a good biomarker for evaluating the prognosis of colorectal tumors, regardless of tumor categorization based on other variables.

### 3.6. Population Screening Detects Colorectal Tumors in Early Stages

**Figure 5:**
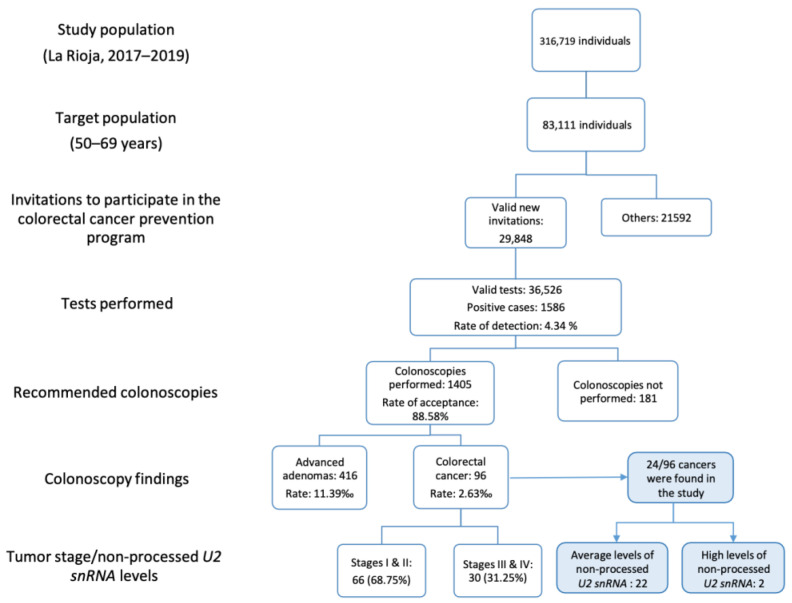
Cross-study of the lack of processing of *U2* snRNA and population screening for colorectal cancer using fecal occult blood tests carried out in La Rioja (Spain) from 2017 to 2019. During the time period evaluated in the study, 83,111 individuals were selected, of which 36,526 completed the fecal occult blood test screening. A total of 1586 had a positive result. Of the 1405 colonoscopies performed, 96 tumors were diagnosed. From 24 colorectal cancer samples, a quantitatively and qualitatively adequate RNA sample was obtained, and the levels of unprocessed *U2* were evaluated. In 22/24 cases, the levels of unprocessed *U2* snRNA were normal and 2/24 cases (8.3%) had high levels of unprocessed *U2* snRNA.

To contrast the social benefit that early detection provides vs. the search for clinicopathological markers that permit doctors to personalize specific tumor treatments, we crossed our results of the impact of snRNA processing in tumors with the population screening carried out in La Rioja (Spain) during the same time period (2017–2019) (Figure 5).

La Rioja is a region in northern Spain, with a population of 316,719 individuals during the years when the study was performed. The colorectal cancer screening program has been deployed in this region since 2010, using fecal occult blood tests. The population screening for early detection of colorectal cancer addressed people aged 50 to 69. (83,111 people) (https://www.ine.es/jaxiT3/Tabla.htm?t=2879&L=0) (accessed on 3 April 2024). Within this target population, an average of 36,526 analyses to detect blood in fecal samples were performed (that is, 44% of the target population, as 56% refused the test for different reasons). A total of 1586 fecal occult blood tests (FOBTs) were positive (4.34% detection rate). As a result of these analyses, 1405 patients with positive results underwent colonoscopies. As expected, a high percentage of patients (88.58%) agreed to undergo a colonoscopy exam once they had the positive blood-in-feces result. Of all the colonoscopies performed, suspicious lesions thought to have a tumor origin were detected in 512 patients. Of these, 416 were advanced adenomas (1.139 % of the people analyzed for blood in their fecal samples) and 96 were diagnosed with colorectal cancer (a rate of 2.63 0/00 of the people analyzed for blood in their fecal samples). Of the patients with colorectal cancer, 66 were grade I or II and 30 were already grade III or IV. These data indicate that colorectal cancer screening is essential to making an early diagnosis. A total of 96 tumors were diagnosed in two years and 68.7% (66/96) of them were in the initial stages of the disease, therefore allowing the cancer to be approached with a curative objective. Finally, 24 of those 96 patients with colorectal cancer came within our study. Of them, 22/24 patients (91.7%) had normal levels of unprocessed *U2* snRNA and 2/24 patients (8.3%) had high levels of unprocessed *U2* snRNA, which are associated with a worse prognosis (Figure 5). Data were obtained from the San Pedro Hospital’s administration (where all the cancer services in the region are centralized) based on a review of the patients’ medical history. These results suggest that the lack of snRNA processing and the consequent accumulation of non-processed snRNAs could be implicated in the quicker progression of the tumors toward higher and more advanced grades.

## 4. Discussion

The Integrator complex has been classically described as being primarily involved in the processing of snRNAs and the pause and release of the RNA pol II at the promoter of specific genes [22]. Mutations in Integrator-complex subunits are known to be involved in several tumors, from prostate cancer [7,8,9], esophageal squamous cell carcinoma [10], nasopharyngeal carcinoma [11], and hepatocellular carcinoma [12,13], to lung carcinoma [14]. While *INTS6* is highly downregulated in most of the tumors and therefore is suggested to play a role as a tumor suppressor, in other tumors, such as colorectal tumor, *INTS6* is markedly upregulated, showing, in this case, an oncogenic activity [47,48].

Lack of snRNA processing and transcriptional profile alteration caused by Integrator-complex deregulation affects the intrinsic molecular mechanism of the tumor transformation of the mutated cells and, therefore, it is independent of its tissular localization. Although some studies indicate that expression of different Integrator-complex subunits, such as INTS6, is altered in specific tumors [13], extensive comparative studies show that INTS6 expression is moderately present in all tissues, being more abundant in the testis, pancreas and liver. In addition, Int6 protein has low cancer specificity. This probably reflects its basic role in cell function, ensuring snRNA maturation and a proper gene expression pattern. Consequently, it is still unclear whether the Int6 protein is a useful tumor biomarker [49]. This apparent paradox (being involved in tumor transformation but having no consistent prognostic value in different tumors) may reflect a tissue-dependent function of Int6 [47,48]. But, additionally, it could be explained at a functional or mechanistic level. In healthy prostate cells, *INTS6* induces cell cycle arrest. Mutations leading to a noticeable reduction in the amount of Int6 protein result in tumor progression. Interestingly, re-expression of *INTS6* cDNA in those tumor cell lines suppresses their high mitosis rate by regulating cell cycle progression [46]. This means that loss of function of *INTS6* is responsible for tumor transformation. In contrast, in colorectal cancer *INTS6* expression is significantly increased when compared with normal tissues [48].

Our results show a positive correlation between levels of unprocessed snRNAs and *INTS6* mRNA in colorectal tumors. This suggests the existence of a feedback mechanism in which *INTS6* expression could be induced upon lack of snRNA processing to revert the accumulation of unprocessed snRNAs. Alternatively, processing of snRNAs could be a finely regulated process and both up- and downregulation of *INTS6* might lead to defects in snRNA processing and readthrough of RNA pol II downstream of the snRNA loci.

In this work, we classified a cohort of colorectal tumors by measuring the degree of snRNA processing and found that the lack of snRNA processing has a better prognostic value than the amount of *INTS6* mRNA. The higher the level of unprocessed snRNA, the worse the survival expectancy for the patient. Our results strengthen the hypothesis that low Integrator-complex activity, but not necessarily low *INTS6* expression, is associated with a poor prognosis in colorectal-cancer patients.

In our cohort we found only tumors that contain normal activity of the Integrator complex (normal processing of snRNAs) or loss of function of the Integrator complex (lack of processing of snRNAs). The fact that we did not find any case in which more efficient processing of snRNAs occurs than in healthy cells indicates that, under normal conditions, the integrator complex is highly efficient in the processing of snRNAs and any mutation or dysregulation unequivocally leads to a loss of function, but never to a gain in function. As expected, other parameters such as high tumor progression or stage show a strong statistical correlation with a poor prognosis.

The transcriptomic changes shown in any of the analyzed tumor groups, either with high or normal processing of snRNAs and low, normal or high levels of *INTS6* mRNA, reveal a notable downregulation of genes involved in standard energy production by mitochondrial respiration, known as the Warburg effect [41,42,43]. This effect was detected in all the groups, indicating that it is a major consequence of tumor transformation. However, it was more notable in the group with high levels of unprocessed snRNAs and low levels of *INTS6* mRNA, suggesting that the Integrator complex might play a direct role in mitochondrial function. It has been shown that, in organisms such as *C. elegans*, *INTS6* controls mitochondrial structure and function [25,26].

The search for biomarkers and the characterization of the molecular mechanism that underlies the tumors is essential for developing efficacious and directed treatments once the tumor is diagnosed. In our work, the accumulation of unprocessed snRNAs as a malignant tumor marker occurs in 19% of all colorectal cancers. However, specific treatments for tumors that present this specific alteration have not yet been established. As a complementary approach, the population screening program for colorectal tumors allows for 94% detection of the tumor transformations in the adenoma phase or early stages (I and II), which can be safely removed by surgical means. This approach, based on early detection, does not discern the molecular mechanism that underlies the tumor transformation. Thus, it is not efficient for treating tumors that are already in the more advanced stages. Both strategies, searching for biomarkers and understanding the molecular mechanisms that underlie the tumors, in combination with prevention and early diagnosis, are complementary for reducing deaths caused by cancer. However, in the short term, cancer-fighting practices based on prevention and early diagnosis have been overwhelmingly more efficient in terms of the broader population.

## 5. Conclusions

In conclusion, in this work we report the clinical significance of the Integrator-complex function in tumor severity and the use of unprocessed snRNA as a biomarker for colorectal cancer prognosis. Larger studies are required to validate the usefulness of unprocessed snRNAs as biomarkers in other types of cancer. Population screening combined with early typing of tumors oriented to identify the molecular mechanisms of their tumor transformation, and development of specific therapies, appear to be the most efficient ways to increase patient survival.

## Figures and Tables

**Table 1:** Pathological characteristics associated with *U2* expression levels. *p*-Values and correlation coefficients.

Variable	Unprocessed *U2* snRNA		Total	*p* Value	*rho* Coefficient
	Normal	High			
All the cases	73	17	90		
Localization within colon	73	17	90	0.951	−0.059
0	24	5	29		
1	28	7	35		
2	21	5	26		
Reason for colonoscopy	73	17	90	0.246	0.124
0	22	2	24		
1	34	12	46		
2	15	3	18		
3	2	0	2		
IHC (Immunohistochemistry)	73	17	90	0.259	−0.135
0	24	6	30		
1	39	10	49		
2	4	0	4		
3	4	1	5		
4	2	0	2		
Adjuvant therapy	72	16	88	0.628	−0.050
0	9	3	12		
1	30	4	34		
2	25	6	31		
3	8	3	11		
Findings in screening colonoscopy	73	17	90	0.130	−0.132
0	36	8	44		
1	20	7	27		
2	13	2	15		
3	4	0	4		
Age	73	17	90	0.591	0.050
<70	23	8	31		
>70	50	9	59		
<75	34	9	43		
≥75	39	8	47		
Colonic structures	73	17	90	0.682	−0.012
0	40	8	48		
1	33	9	42		
Anatomical Pathology	73	17	90	0.054	0.213 (*)
0	3	0	3		
1	66	15	81		
2	4	2	6		
CEA (Carcinoembryonic antigen)	73	17	90	0.855	−0.002
Progression	72	16	88	0.653	0.044
0	47	8	56		
1	25	8	33		
Concomitant diagnosis of other tumors	72	16	88	0.484	0.078
0	60	13	73		
1	12	3	15		
Survivorship	73	16	89	0.029	−0.229 (*)
Tumor stage	73	17	90	0.594	0.050
0	15	2	17		
1	16	3	19		
2	26	8	34		
3	16	4	20		
INTS6 mRNA	73	17	90	0.000	0.436 (**)
0	12	4	16		
1	29	1	30		
2	32	12	44		

*. The correlation is significant at the 0.05 level (two-tailed). **. The correlation is significant at the 0.01 level (two-tailed).

**Table 2:** Classification of the analyzed colorectal tumor samples based on *U2* snRNA processing and *INTS6* mRNA levels. Numbers correspond to biopsy samples. Samples were grouped in the different categories depending on whether the optical density values of the semi-quantitative RT-PCRs gel bands were high (greater than the mean + SD of the controls), medium (between the mean ± SD of the controls) or low (less than the mean − SD of the controls).

	LOW Levels of *INTS6*	NORMAL Levels of *INTS6*	HIGH Levels of *INTS6*
NORMAL levels of non-processed *U2*	1, 15, 18, 19, 20, 25, 28, 29, 48, 50, 175, 176	72, 107, 111, 131, 137, 138, 140, 146, 160, 161, 166, 167, 168, 173, 174, 180, 182, 183, 184, 187, 188, 189, 190, 194, 195, 197, 201, 202, 203	23, 40, 45, 54, 55, 58, 60, 62, 67, 68, 71, 73, 79, 80, 101, 105, 115, 118, 121, 122, 124, 129, 141, 164, 169, 171, 172, 179, 191, 193, 199, 200
HIGH levels of non-processed *U2*	17, 21, 32, 33	119	22, 35, 37, 38, 41, 43, 56, 75, 90, 94, 120, 185

## Data Availability

Raw sequence data generated in this study are available in the Gene Expression Omnibus (GEO) data repository (https://www.ncbi.nlm.nih.gov/geo/) (Accession number GSE261888).

## Supplementary Materials

The following supporting information can be downloaded at: https://www.mdpi.com/article/10.3390/cancers16132340/s1, Figure S1: Graphical representation of the multivariate analysis of the age, tumor progression and *INTS6* mRNA value variables with respect to the unprocessed *U2* snRNA values; Table S1: Differentially Expressed Genes (DEGs) of each tumor group based on *INTS6* levels and snRNA processing compared to healthy individual biopsies; Table S2: Gene Ontology (GO)-term enrichment within the Differentially Expressed Genes (DEGs) of each tumor group based on *INTS6* levels and snRNA processing compared to healthy individual biopsies; Table S3: transformation of categorical variables into ordinal scales; Table S4: Values of the *rho* coefficients in the correlation matrix and *p* values in paired sample correlation (Student’s *t*-test distribution); Table S5: Univariate and multivariate Cox analyses.
