# Supplementary material for: Unprocessed snRNAs Are a Prognostic Biomarker and Correlate with a Poorer Prognosis in Colorectal Cancer
Source: Cancers (Basel). 2024 Jun 26;16(13):2340. doi: 10.3390/cancers16132340 (PMC11240374; doi:10.3390/cancers16132340)

U2 snRNA	Age	Progression	INT6		
U2=normal U2=high	72,4111	Yes	Low	Medium	High

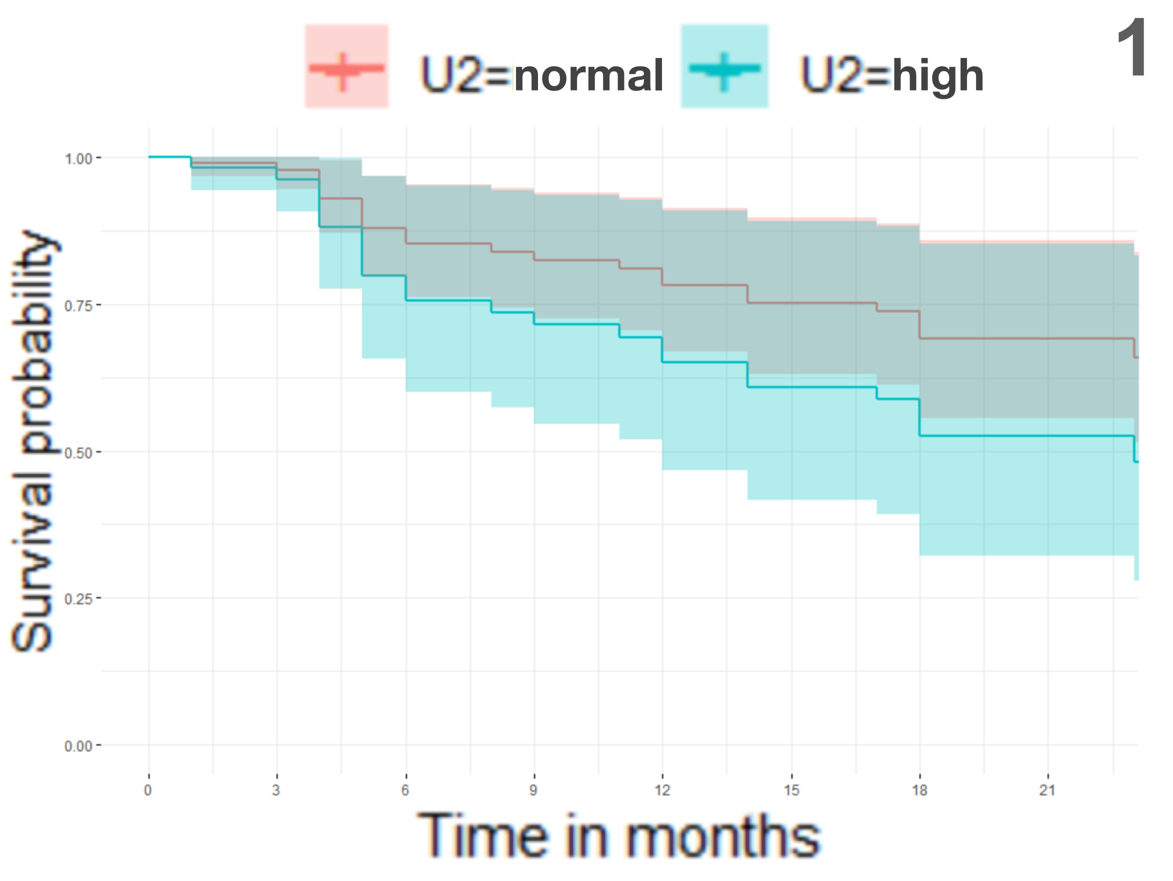

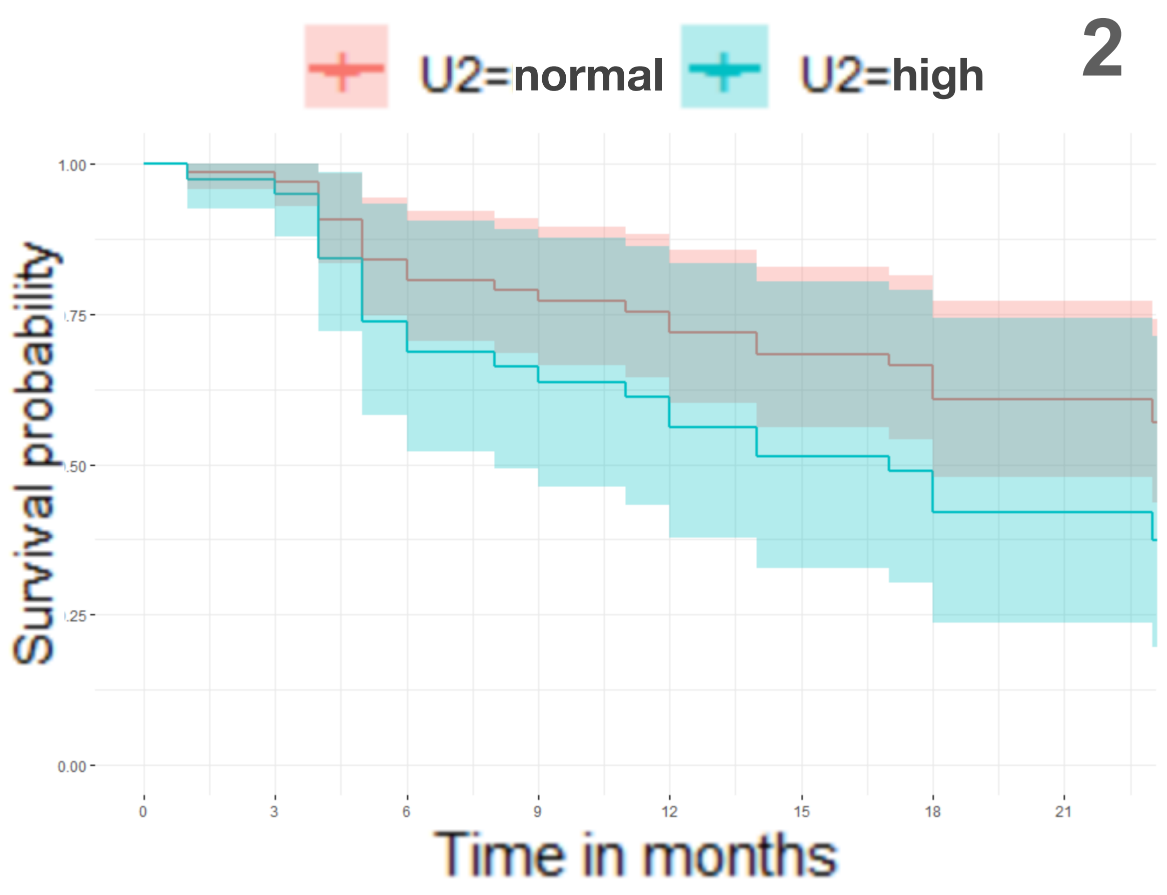

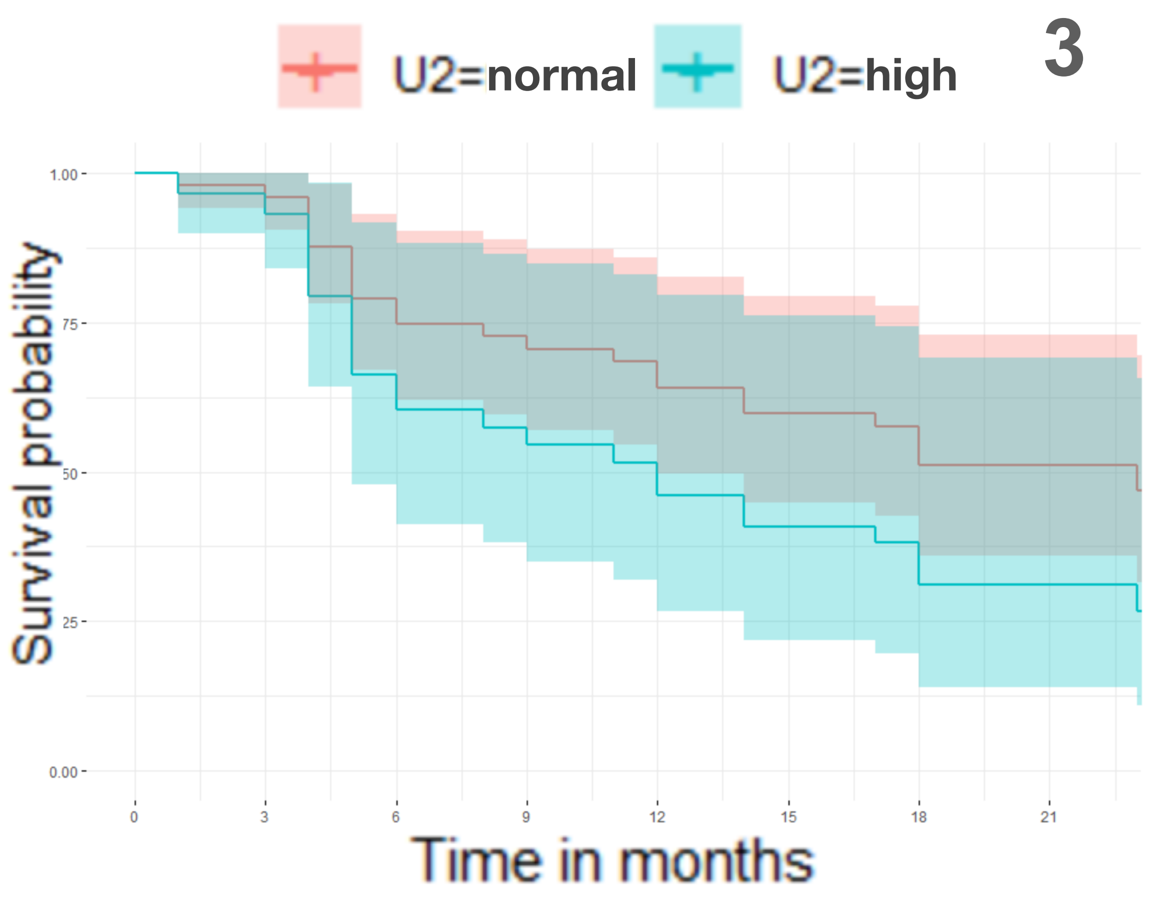

U2 snRNA	Age	Progression	INT6		
U2=normal U2=high	72,4111	No	Low	Medium	High

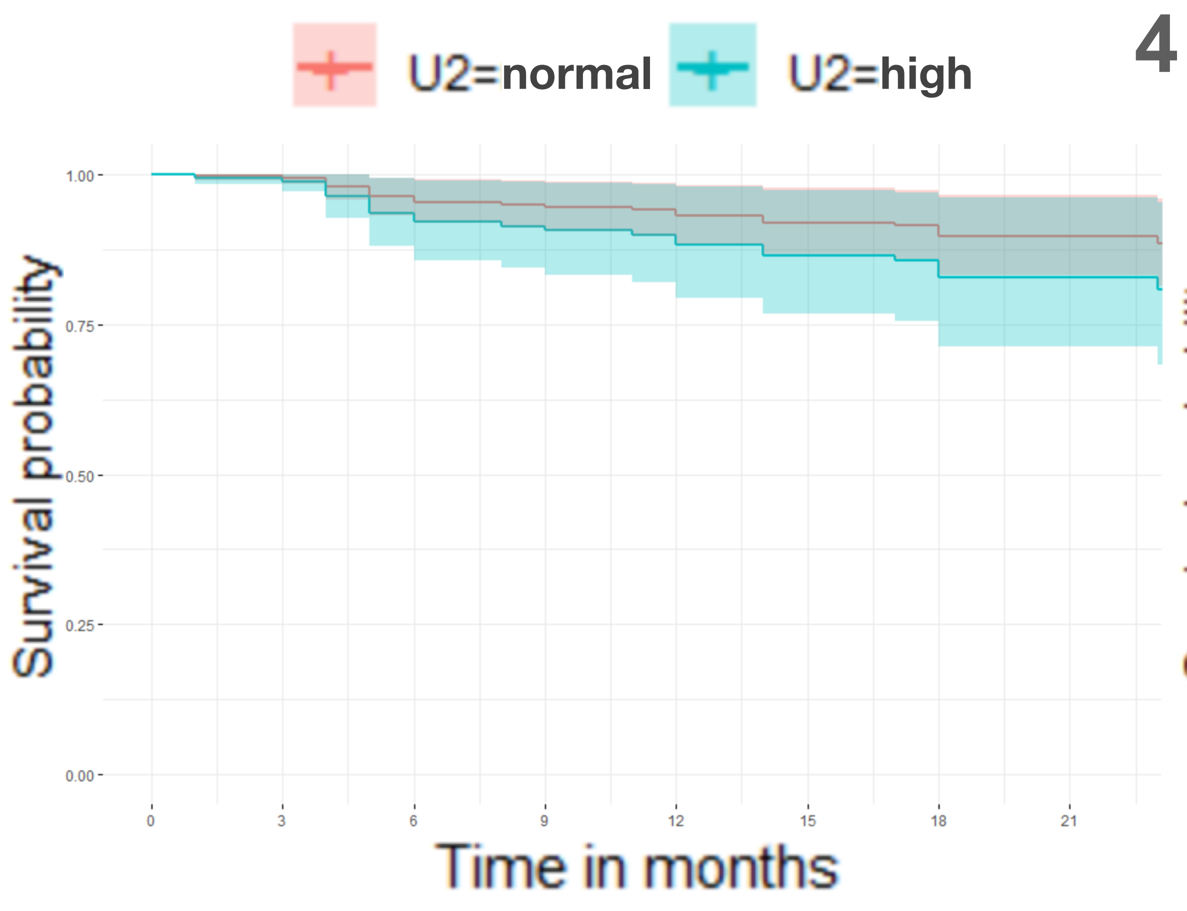

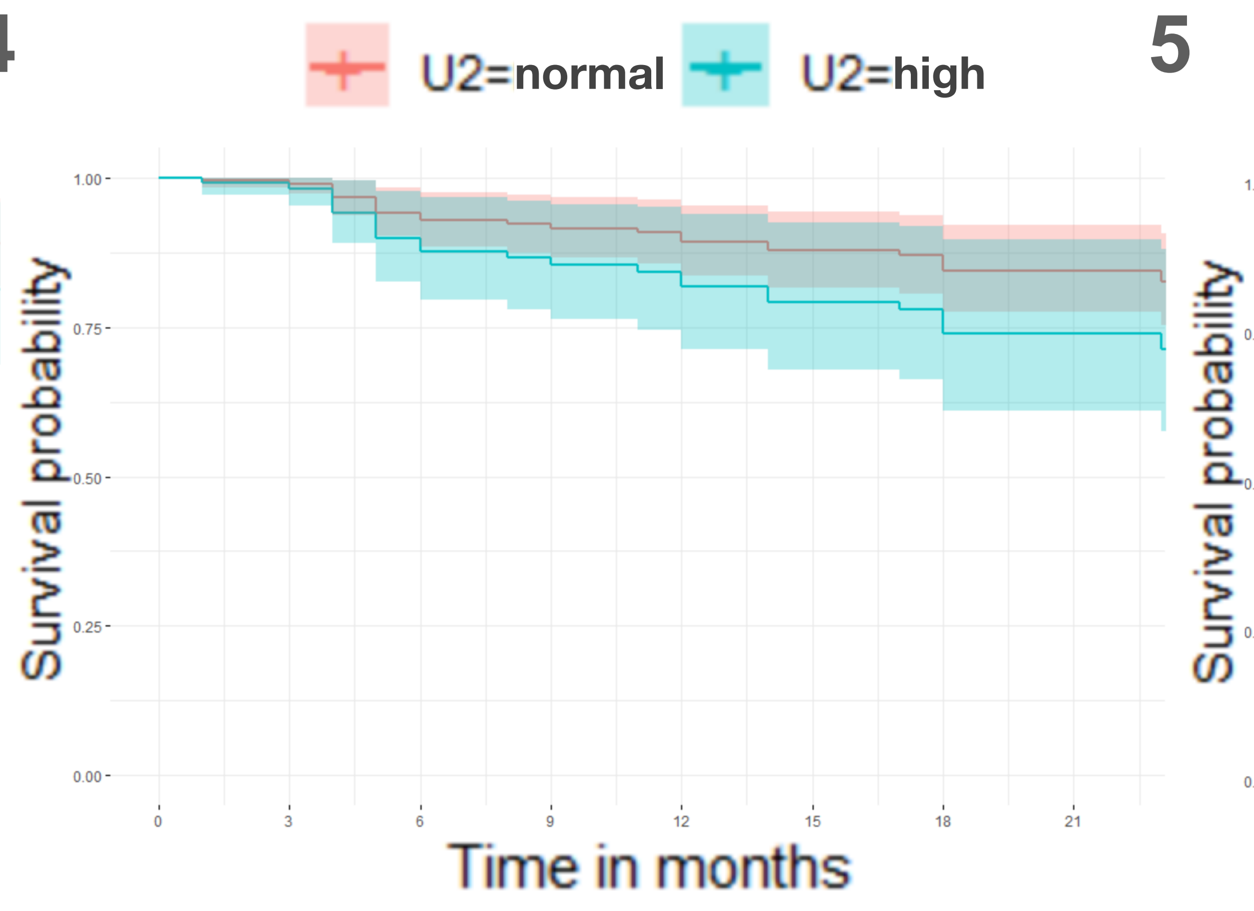

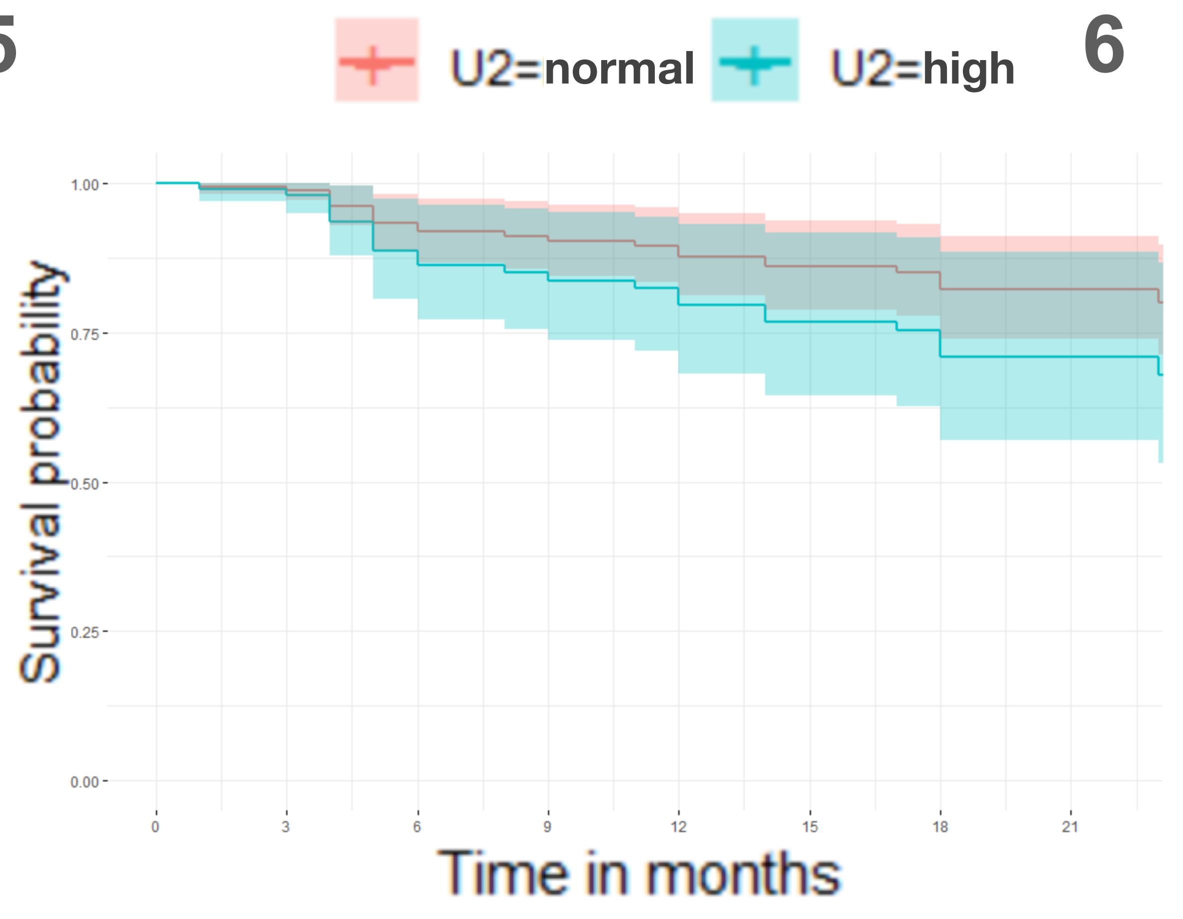

Supplement: Supplementary file 1 [file cancers-16-02340-s001.zip › Figure_S1.pdf]
