# Supplementary material for: Unprocessed snRNAs Are a Prognostic Biomarker and Correlate with a Poorer Prognosis in Colorectal Cancer
Source: Cancers (Basel). 2024 Jun 26;16(13):2340. doi: 10.3390/cancers16132340 (PMC11240374; doi:10.3390/cancers16132340)
Supplement: Supplementary file 1 [file cancers-16-02340-s001.zip › sup. legends.docx]

**Figure S1:**

Graphical representation of the multivariate analysis of tumor progression and *INT6* mRNA value variables with respect to the unprocessed U2 snRNA values.

Graph 1 shows the effect of snRNA processing on survival with tumor progression and low levels of *INTS6*.

Graph 2 shows the effect of snRNA processing on survival with tumor progression and medium levels of *INTS6*.

Graph 3 shows the effect of snRNA processing on survival with tumor progression and high levels of *INTS6*.

Graph 4 shows the effect of snRNA processing on survival without tumor progression and low levels of *INTS6*.

Graph 5 shows the effect of snRNA processing on survival without tumor progression and low levels of *INTS6*.

Graph 6 shows the effect of snRNA processing on survival without tumor progression and low levels of *INTS6*.

With disease progression and high levels of unprocessed U2 snRNA, the probabilities of survival are close to 35% at 18 months, while with normal values of unprocessed U2 snRNA, survival increases to 52%. Without disease progression, with high levels of unprocessed U2 snRNA and high INTS6 mRNA levels, the probabilities of survival are close to 75%.

**Table S1:**

Differentially Expressed Genes (DEG) of each tumor group based on *INTS6* levels and snRNA processing compared to healthy individual biopsies.

Group 1: Normal *U2* low *INTS6*

Group 2: Normal *U2* normal *INTS6*

Group 3: Normal *U2* high *INTS6*

Group 4: High unprocessed *U2* low *INTS6*

Group 5: High unprocessed *U2* low *INTS6* (only found one tumor in the cohort.

Group 6: High unprocessed *U2* high *INTS6*

Group 5 was not analyzed because there was only one tumor falling in this category in the cohort. Statistical analysis was not possible.

**Table S2:**

Gene Ontology (GO) terms enrichment within the Differentially Expressed Genes (DEG) of each tumor group based on *INTS6* levels and snRNA processing compared to healthy individual biopsies.

Group 1: Normal *U2* low *INTS6*

Group 2: Normal *U2* normal *INTS6*

Group 3: Normal *U2* high *INTS6*

Group 4: High unprocessed *U2* low *INTS6*

Group 5: High unprocessed *U2* low *INTS6* (only found one tumor in the cohort.

Group 6: High unprocessed *U2* high *INTS6*

**Table S3:**

Clinicopahological variables transformed into ordinal variables for analysis purposes. Each category within the original variables has been assigned a numerical value based on its meaningful order or ranking.

**Table S4:**

*rho* coefficients and p values in paired samples correlation (t-Student distribution).

**Table S5:**

Univariate and multivariate Cox analysis.

(A) Table shows the univariate analysis. The tumor stage, findings in the screening colonoscopy and tumor progression variables have highly statistically significant coefficients, while the other variables do not.

The beta coefficients for tumor stage and progression are positive, while the findings in the screening colonoscopy are negative. Therefore, advanced stage and advanced progression are associated with lower survival, while high values on screening colonoscopy, which imply early detection of polyps or recurrence, are associated with better survival.

B) In the multivariate analysis, we chose values with the highest hazard ratio and age. The age variable has a p value of 0.09352, with a risk index HR=1.03, and a 95% confidence interval of 0.015-1.677). These results indicate that age makes a minor contribution to the difference in function after adjusting the progression and U2 values.
